# Cilia in the choroid plexus: their roles in hydrocephalus and beyond

**DOI:** 10.3389/fncel.2015.00039

**Published:** 2015-02-12

**Authors:** Keishi Narita, Sen Takeda

**Affiliations:** Department of Anatomy and Cell Biology, Interdisciplinary Graduate School of Medicine and Engineering, University of YamanashiChuo, Yamanashi, Japan

**Keywords:** cilia, diversity, hydrocephalus, multiciliogenesis, cerebrospinal fluid

## Abstract

Cilia are whip-like projections that are widely conserved in eukaryotes and function as a motile propeller and/or sensory platform to detect various extracellular stimuli. In vertebrates, cilia are ubiquitously found in most cells, showing structural and functional diversities depending on the cell type. In this review, we focus on the structure and function of cilia in choroid plexus epithelial cells (CPECs). CPECs form one or two dozen non-motile 9+0 cilia, which display transient acquisition of motility during development. Genetic malfunction of cilia can lead to failure of multiple organs including the brain. Especially, several groups have demonstrated that the defects in CPEC cilia cause the communicating form of hydrocephalus. In order to elucidate the molecular mechanisms underlying the hydrocephalus, we have previously demonstrated that the cilia possess an NPFF receptor for autocrine signaling to regulate transepithelial fluid transport. In this perspective, we also discuss the potential involvement of cilia in the other aspects of choroid plexus functions, such as the regulation of brain development and neuroinflammation.

## Overview of vertebrate cilia

**Figure 1:**
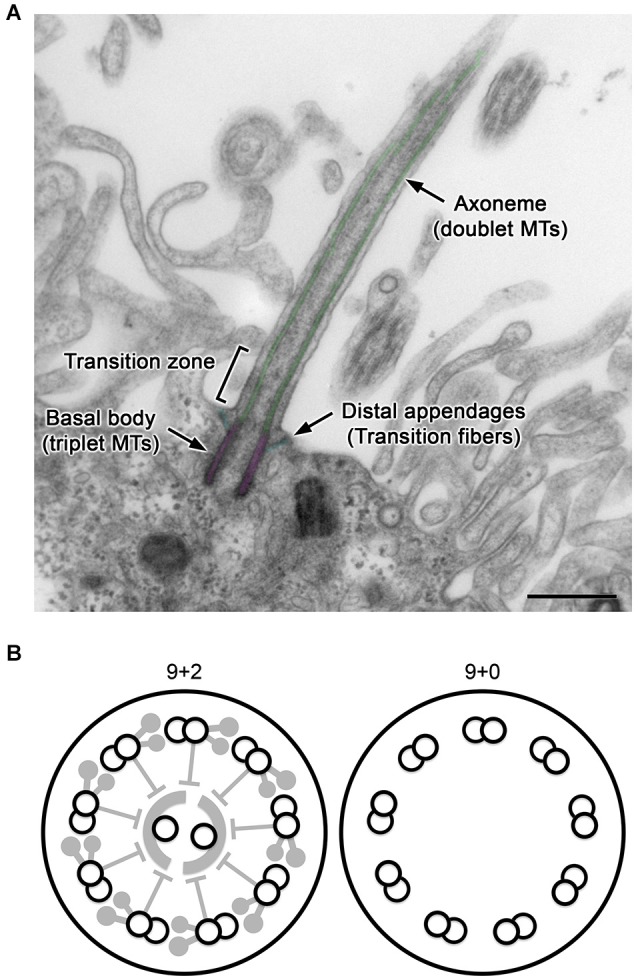
**General structure of cilia. (A)** A longitudinal section of a CPEC cilium. The cilium emerges from the microvilli-rich apical cell surface. The structure is supported by the axoneme and basal body (pseudo-colored in green and magenta, respectively). The distal appendage (cyan) connects to the basal body and cell membrane. In the transition zone, characteristic Y-shaped structures bridges the axoneme and ciliary membrane, which can be recognized in horizontal sections. MTs, microtubules. Bar, 500 nm. **(B)** Schematics of transverse sections of motile 9+2 and non-motile 9+0 cilia.

Cilia are hair-like projections on the cell surface with a diameter of ~250 nm and various lengths of typically 5–10 μm (Figure 1A). Their structure is supported and anchored to the cell by characteristic cytoskeletal scaffolds called the axoneme and basal body in which doublet and triplet microtubules, respectively, are radially arranged with nine-fold symmetry. Cilia are widely conserved across eukaryotic species, and in many unicellular organisms, their active vibration is necessary for propelling the cell. In vertebrates, cilia have been observed with various characteristics, such as length, motility, and number per cell, depending on the tissues and cell type including neurons and glia in the brain (Gerdes et al., 2009; Louvi and Grove, 2011; Takeda and Narita, 2012).

For example, ependyma (ependymocytes) lining brain ventricles form hundreds of motile cilia to circulate the cerebrospinal fluid (CSF). The axoneme of this ciliary subtype has a central pair of singlet microtubules (termed “9+2”), and is heavily equipped with axonemal dyneins and their regulatory complexes, which collectively drive the back-and-forth movement of cilia (Figure 1B, left; Afzelius, 2004; Lindemann and Lesich, 2010). In contrast, most neurons and glia possess solitary non-motile cilia called primary cilia. Their axoneme has no central pair and is termed “9+0” (Figure 1B, right). Compared with 9+2 cilia (Heuser et al., 2009; Pigino et al., 2011), the structural details have been poorly resolved in 9+0 cilia (Gilliam et al., 2012). Although most primary cilia appear to lack axonemal dyneins and are non-motile except for nodal cilia (Takeda et al., 1999; Hirokawa et al., 2009), they harbor various cell signaling receptors and mediators to detect and process mechanical stress or chemical stimuli such as Sonic hedgehog and platelet-derived growth factor (Praetorius and Spring, 2001; Corbit et al., 2005; Schneider et al., 2005; Yoshimura et al., 2011; Briscoe and Thérond, 2013; Su et al., 2013). The outer segment of photoreceptors in the retina, where photosensitive rhodopsins are packed in a series of membranous discs, is a specialized form of primary cilia (Gilliam et al., 2012).

Genetic defects leading to ciliary malfunctions cause disorders with clinically variable phenotypes. Such disorders are called ciliopathies and include primary ciliary dyskinesia, polycystic kidney disease, Leber congenital amaurosis, nephronophthisis, Senior-Løken syndrome, Joubert syndrome, Bardet-Biedl syndrome, and Meckel Gruber syndrome (Novarino et al., 2011). These ciliopathies are often associated with brain diseases such as neural tube defects, cerebellar hypoplasia, mental retardation, and hydrocephalus.

## Biogenesis of cilia

Numerous studies using various model organisms, such as green algae, worms, fish, frogs, and mice, as well as human subjects, have founded the principle of ciliogenesis as recapitulated below. This information also provides the basis to understand ciliopathies.

The biogenesis of cilia is initiated by assembling axonemes and docking ciliary membrane vesicles to the distal end of the basal body. A specialized transport system called “intraflagellar transport” (IFT) carries tubulin and other materials along the axoneme (Rosenbaum and Witman, 2002). IFT facilitates microtubule motor proteins, kinesins and cytoplasmic dyneins, as well as IFT particles A and B, which mediate cargo attachment to the motors. For docking to the ciliary membrane, structural components of the appendages on the basal body are required (Tanos et al., 2013; Veleri et al., 2014). Furthermore, the regulation of molecules entering and leaving cilia is mediated by several other systems including the BBSome (Nachury et al., 2010). A molecular sieve and septin ring at the ciliary base also restrict simple diffusion of soluble and membrane proteins, respectively (Hu et al., 2010; Breslow et al., 2013; Lin et al., 2013).

The basal body of a primary cilium is a modified mother centriole. When cells enter the cell cycle, the primary cilium is shortened, and the basal body is detached from the cell surface to function as a microtubule-organizing center (Paridaen et al., 2013). On the other hand, the basal bodies of multiciliated cells are generated explosively de novo at intracellular foci called deuterosomes by the so-called acentriolar pathway (Klos Dehring et al., 2013). Foxj1 is one of the transcription factors that act as a master regulator of multiciliogenesis (Thomas et al., 2010).

## Cilia in choroid plexus epithelial cells

The choroid plexus is a highly undulating and vascularized tissue that protrudes into brain ventricles. Its epithelium consists of choroid plexus epithelial cells (CPECs) that produce CSF with high efficiency (Damkier et al., 2013). In addition, the choroid plexus epithelium secretes ligands that are important for brain physiology, and regulates protein diffusion and leukocyte infiltration from systemic circulation (Redzic et al., 2005; Reboldi et al., 2009; Shechter et al., 2013). Because CPECs are derived from the dorsal neuroepithelium and form a continuous monolayer with ependyma, they are sometimes described as choroidal or modified ependyma. However, CPECs and ependyma are distinct in many aspects, which is also the case for cilia.

**Figure 2:**
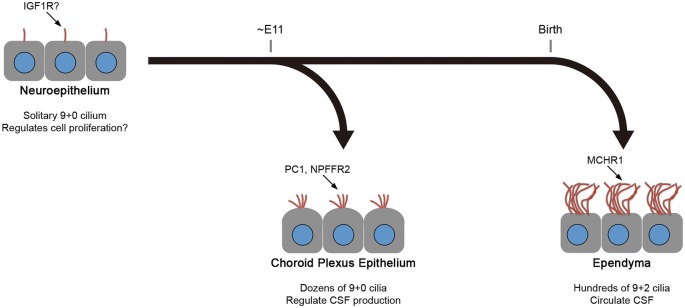
**Differences between CPEC and ependymal cilia**. The formation of multiple cilia in CPECs occurs shortly after the cells differentiate from the neuroepithelium during organogenesis (about embryonic day 11 in mice). The cilia exhibit transient motility during the perinatal period, which peaks at the day of birth, and eventually become non-motile. However, ependyma undergo multiciliogenesis after birth to establish hundreds of motile cilia in 2 weeks. The beating orientation is aligned at both cellular and tissue levels by planar cell polarity signaling. In both cell types, multiciliogenesis is associated with the induction of transcription factors, FOXJ1 and RFX3. Ciliary localization of indicated molecules in neuroepithelium, choroid plexus epithelium, and ependyma are reported or implicated in Lehtinen et al. (2011), (Banizs et al., 2005; Wodarczyk et al., 2009; Narita et al., 2010), and Conductier et al. (2013), respectively. Knockout mice lacking general ciliogenesis genes, such as *Ift88*, *Kif3a*, and *Bbs1*,*2*,*4*, and *6*, exhibit the communicating form of hydrocephalus. See text for details.

As described above, mature ependyma form hundreds of motile 9+2 cilia that beat in a concerted manner to circulate CSF. In mouse, the multiciliogenesis initiates after birth and requires about 2 weeks for full maturation (Figure 2; Spassky et al., 2005). In contrast, CPECs form one or two dozen non-motile 9+0 cilia (Narita et al., 2010). Ciliogenesis in CPECs occurs shortly after the choroid plexus primordia begins to bud during organogenesis (Figure 2; Nonami et al., 2013). In addition, CPEC cilia exhibit transient motility around the perinatal period, yet a low beating frequency, small amplitude, and random orientation are all unfavorable to generate directional CSF flow (Narita et al., 2012). The motility peaks at around the day of birth and declines progressively during the following 2 weeks. While both CPECs and ependyma may share a common, FOXJ1-dependent mechanism to initiate multiciliogenesis (Lim et al., 1997; Narita et al., 2012), their cilia show different characteristics. This observation is intriguing from the viewpoint of the current principle.

Genetically modified mouse models have also shown differences in the mechanism of ciliary formation and/or the maintenance of cilia in CPECs and ependyma. In a knockout mouse for *Celsr2*, an ortholog of the planar cell polarity gene *Flamingo*, an impairment of ciliogenesis is observed in ependyma but not in CPECs (Tissir et al., 2010). Similarly, forced expression of the PAC1 (phosphatase of activated cells 1) receptor, a G protein-coupled receptor that is predominantly expressed in the central nervous system (CNS) and selectively activated by pituitary adenylate cyclase-activating polypeptide, causes severe hydrocephalus associated with disorganization of ependymal cilia, while CPEC cilia are unaffected (Lang et al., 2006).

Regarding the unique function of CPEC cilia, several groups including ours have reported the potential involvement of CPEC cilia in the regulation of CSF production. Analysis of CPEC cilia in relation to the hydrocephalus was first described by Yoder et al. (Banizs et al., 2005). In the *Ift88^Tg737Rpw^* mouse that has defects in IFT88 expression and function, Banizs et al. observed a communicating form of the hydrocephalus at neonatal periods, when most ependyma lack mature motile cilia. During these stages, CPEC cilia show an accumulation of polycystin-1, the defects of which cause autosomal dominant polycystic kidney disease, in a bulb-like structure at the tip. This abnormal ciliary structure and protein localization coincide with an increase in cellular cAMP levels and aberrant regulation of intracellular pH and ion transport activities in CPECs (Banizs et al., 2005, 2007). Similarly, Wodarczyk et al. also described the ciliary localization of polycystin-1 in CPECs and ependyma (Wodarczyk et al., 2009). They generated ubiquitous or brain-specific *Pkd1* knockout mice, which encodes polycystin-1, and observed hydrocephalus at perinatal periods in both mouse lines.

We used a primary culture system for swine CPECs to analyze ciliary function and showed that deciliation by chloral hydrate increases both intracellular cAMP levels and basolateral-to-apical transepithelial fluid transcytosis, which is consistent with the above observations by Banizs et al. (Narita et al., 2010). We also demonstrated localization of neuropeptide FF receptor 2 on CPEC cilia, and its autoactivation downregulated cellular cAMP levels and fluid transcytosis. While the mechanism involves negative regulation of CSF production, we do not know whether there is a positive regulator or the production is sustained continuously, and only negative regulation controls the amount of CSF (Lindvall et al., 1978; Damkier et al., 2013). This point has to be addressed in the near future.

Swiderski et al. investigated the mechanism of ventriculomegaly that is common in ciliopathy models of *Bbs1*, *Bbs2*, *Bbs4*, and *Bbs6* mutant mice (Swiderski et al., 2012). While ventriculomegaly is not associated with stenosis of the cerebral aqueduct, ultrastructural abnormalities in the cilia of CPECs, ependyma, and some circumventricular organs are observed consistently in these mutant mice at various ages. The previous study also concluded that a loss of regulation in CSF production is one of the possible mechanisms underlying the pathology.

Recently, Liu et al. generated a conditional knockout of *Kif3a* in cranial neural crest cells, using a Wnt1 promoter-driven Cre recombinase (Liu et al., 2014). KIF3A is a kinesin motor protein involved in ciliogenesis and plays a crucial role in the determination of left-right asymmetry of the body (Takeda et al., 1999). The genetically modified mice exhibited ciliopathy phenotypes of craniofacial anomalies and hydrocephalus. Regarding the hydrocephalus, the authors observed a dramatic dilation of the lateral and third ventricles in E16.5 embryo. Having confirmed the Wnt1*^cre^* expression in E16.5 choroid plexuses, they concluded that the hydrocephalus is due to overproduction of CSF (Liu et al., 2014).

## Future perspectives

The above studies implicate defects in CPEC cilia as a cause of the communicating form of hydrocephalus. However, reports by Durand et al. suggest additional mechanisms. They generated mice deficient for Rfx3, a transcription factor that regulates ciliogenesis, and demonstrated marked inhibition of ciliogenesis in both CPECs and ependyma, which is associated with the communicating form of hydrocephalus (Baas et al., 2006; El Zein et al., 2009) and in agreement with the above studies. Interestingly, the authors also observed marked choroid plexus hypogenesis in the knockout mouse (Baas et al., 2006; El Zein et al., 2009). Because CPECs synthesize and secrete various growth factors and signaling molecules for brain development (Redzic et al., 2005; Reboldi et al., 2009; Shechter et al., 2013), a reduction in the mass of choroid plexus tissue may lead to brain abnormalities other than hydrocephalus. Indeed, the authors later demonstrated that the *Rfx3* knockout mouse also exhibits corpus callosum agenesis (Benadiba et al., 2012), although the significance of choroid plexus hypogenesis in this phenotype is unclear.

A growing body of evidence suggests that the choroid plexus functions as a selective and educative gate for circulating immune cells in the immune surveillance of the CNS to resolve neuroinflammation under pathological conditions (Schwartz and Baruch, 2014). The apical surface of CPECs is the site where immune cells reside even under physiological conditions. These cells were initially described as epiplexus cells or Kolmer cells (Ling et al., 1998), and are now recognized as dendritic cells and macrophages that function as local antigen-presenting cells (Ransohoff and Engelhardt, 2012). When activated by inflammatory cytokines, CPECs upregulate their expression of integrin receptors to promote immune cells entering the CNS (Engelhardt et al., 2001; Shechter et al., 2013). Because of the physical proximity, it is possible that CPEC cilia make direct contact with these immune cells and/or receive chemical substances secreted by them, thereby participating in the regulation of choroid plexus functions in response to neuroinflammation.

Recently, we performed proteomic analysis of CPEC cilia from swine and identified >800 proteins (Narita et al., 2012). Among them, 45% were shared with the proteome of the 9+0 photoreceptor outer segment and 18% were shared with the proteome of 9+2 cilia and flagella. Among the remaining 37% of the proteins including the CPEC-specific ciliome subset, various signaling molecules were enriched. Functional analysis of these proteins will clarify the role of CPEC cilia in more detail and their link to brain disorders.

According to the traditional view, CPECs have been regarded as solely responsible for the production of CSF. However, based on our current understanding of CSF production, we should re-interpret or re-evaluate the traditional views of CSF homeostasis (Iliff et al., 2012), which are not necessarily obsolete or invalid. In this regard, the cilia in CPECs may have various unknown functions that are related to maintenance of brain homeostasis. Therefore, cilia in the brain ventricular system play important biological roles in neurophysiology and may further advance our understanding of brain functions.

## Conflict of interest statement

The authors declare that the research was conducted in the absence of any commercial or financial relationships that could be construed as a potential conflict of interest.
